# The effect of stress mindset on psychological pain: the chain mediating roles of cognitive reappraisal and self-identity

**DOI:** 10.3389/fpsyg.2025.1517522

**Published:** 2025-03-19

**Authors:** Shun Qiang, Jinxia Wu, Dewei Zheng, Tingting Xu, Yangkun Hou, Jianlong Wen, Jianlan Liu

**Affiliations:** ^1^School of Clinical Medicine, Shandong Second Medical University, Weifang, Shandong, China; ^2^School of Psychology, Shandong Second Medical University, Weifang, Shandong, China; ^3^School of Public Health, Shandong Second Medical University, Weifang, Shandong, China

**Keywords:** stress mindset, psychological pain, self-identity, cognitive reappraisal, medical students

## Abstract

**Background:**

Psychological pain is the most important factor affecting suicide rates. However, the factors contributing to psychological pain in medical students remain poorly understood.

**Objective:**

This study aimed to investigate the effects of a stress mindset on psychological pain and the mediating roles of cognitive reappraisal and self-identity among Chinese medical students.

**Methods:**

Medical students (*N* = 2056; 1,369, 66.60% female; 683, 33.40% male) from China completed multiple measures of stress mindset, self-identity, cognitive reappraisal, and psychological pain. Correlation and mediation analyses were conducted using SPSS 29.0 and Mplus 8.3.

**Results:**

(1) The stress mindset was significantly negatively correlated with psychological pain among medical students. (2) Cognitive reappraisal mediated the relationship between the stress mindset and psychological pain. (3) Self-identity mediated the relationship between the stress mindset and psychological pain. (4) Cognitive reappraisal and self-identity jointly mediated the relationship between the stress mindset and psychological pain.

**Conclusion:**

The results of this study deepen the understanding of the psychological processes linking stress mindset and psychological pain and provide a valuable reference for reducing psychological pain and improving the well-being of medical students.

## 1 Introduction

### 1.1 Stress mindset and psychological pain

Medicine has always been regarded as a challenging discipline. Due to the many inherent disadvantages of modern medical education, medical students suffer from significant psychological stress during their growth and learning process, such as the rigor of the course content, which often makes students feel exhausted, leading to an increased risk of insomnia as well as depression (Dyrbye et al., 2006). In addition, in clinical practice, medical students often face inequalities in status and power, and are particularly disadvantaged in their interactions with clinical teaching faculty and hospital management (Moczko et al., 2016; Rich, 2023). At the same time, medical students must empathize with patients while acquiring specialized knowledge in hospitals, resulting in an emotional burden (Rieffestahl et al., 2021). This status quo is likely to contribute to the psychological pain experienced by medical students.

Shneidman defines psychological pain as an acute state of intense distress associated with feelings of guilt, fear, panic, anxiety, loneliness, and helplessness (Shneidman, 1993). In contrast to physical pain, which is an immediate emotional response to stressful life events, psychological pain is a subjectively widespread, intense, and complex emotional experience centered on a sense of distress resulting from negative self-perception and ineffective coping strategies based on personality traits. Psychological pain has serious implications; it leads to a decline in the academic performance of medical students and can carry over into their future careers, affecting the patient-physician relationship and the quality of healthcare services provided (Meredith et al., 2022). In addition, psychological pain can weaken an individual’s ability to cope effectively with life’s difficulties and stresses, leading them to view suicide as a means of escape from reality (Baryshnikov and Isometsä, 2022; Chen et al., 2023). Furthermore, many studies have shown that psychological pain is a significant predictor of suicide risk (Rizvi et al., 2017). In China, Confucian culture emphasizes self-restraint, the suppression of emotions such as happiness and anger, hierarchical relationships between elders and juniors, and distinctions between respect and inferiority (Huang et al., 2024). The stigma surrounding mental health issues may lead medical students to underreport or conceal their psychological pain, creating barriers to self-disclosure and help-seeking. This cultural influence shapes how medical students perceive and report psychological pain, potentially leading to an underestimation of their emotional struggles. Relevant studies have shown that medical students in mainland China have a high rate of suicidal ideation (Zeng et al., 2019; Huang et al., 2024). This suggests the need to study psychological pain among Chinese medical students and its influencing factors to improve their mental health.

According to implicit theories, mindsets can affect an individual’s understanding and judgment of information. Mindsets can thereby reduce or exacerbate the impact of stress on an individual’s emotional, behavioral, and physiological responses, leading to fluctuations in levels of anxiety, depression, and other undesirable emotions (Burnette et al., 2013; Schreiber and Schotanus-Dijkstra, 2024). Crum proposed the concept of a stress mindset, which refers to how an individual perceives a stressful event. Individuals who believe that stress can improve their cognitive performance, mental health, and other outcomes have a stress-is-enhancing mindset (SEM). Conversely, individuals who believe that stress has a negative effect on these outcomes have a stress-is-debilitating mindset (SDM)(Crum et al., 2013). Stress mindset is a continuous process. Neurophysiological evidence suggests that interventions in the cognitive processes associated with psychological pain alter the intensity of pain perception (Flor, 2014). One study pointed out that a more positive stress mindset can reduce traumatic stress symptoms after an individual experiences a traumatic life event (Williams and Ginty, 2024). Another study showed that a SEM reduced depression and anxiety symptoms in college students in high-stress environments, whereas college students with a SDM were more likely to experience depression and burnout after an increase in stressful events (Huebschmann and Sheets, 2020). In other words, a stress mindset may influence people’s levels of psychological pain, with a SEM contributing to a reduction in an individual’s level of psychological pain, while a SDM exacerbates psychological pain.

### 1.2 The effect of stress mindset on psychological pain: the mediating role of cognitive reappraisal

The emotion regulation theory emphasizes the variety of regulatory strategies that individuals adopt when dealing with emotions. Different emotion regulation strategies can produce different beneficial or harmful results. These strategies are the means and methods that individuals use to manage and adjust their emotions, helping them cope with emotional distress, reducing the impact of negative emotions, and increasing positive emotional experiences (Gross, 2015). Cognitive reappraisal and expression suppression are the two main emotion regulation strategies. The former initiates emotion regulation at an early stage of emotion generation by changing the cognitive appraisal of the emotion-eliciting situation, whereas the latter involves active suppression of emotions after they have been generated. These two strategies are relatively independent and have distinct neural bases (Sun et al., 2020). Research has demonstrated that cognitive reappraisal is more effective in emotion regulation than the suppression of expression (Goldin et al., 2008; Wu et al., 2019). Individuals who tend to use cognitive reappraisal strategies are more likely to have a favorable view of their circumstances and feel more positive, happy, and joyful (Chirico et al., 2021; Vanderlind et al., 2022). In addition, Crum et al. (2017) noted that people with a SEM are better at adjusting their cognition; therefore, they are more confident when facing difficulties and better at using cognitive reappraisal strategies.

### 1.3 The effect of stress mindset on psychological pain: the mediating role of self-identity

Self-identity refers to an individual’s cognitive understanding and definition of themselves, including their identity, values, beliefs, goals, and roles in society (Gu et al., 2022). It deals with how individuals perceive and position themselves in various social situations. It creates a sense of harmony and coherence within the individual and helps unify self-concepts (Arnett, 2023). According to cognitive behavioral theory, an individual’s cognition influences emotions and behavior. Individuals with positive self-recognition are likely to experience positive emotions. When individuals experience self-confusing negativity, it can lead to denial of themselves and the formation of psychological pain. A study indicated that adolescents with a high sense of identity had higher levels of psychosocial adjustment, self-confidence, and happiness (Meeus et al., 2010; Hatano et al., 2018). Conversely, individuals with a low sense of identity exhibit higher levels of aggressive behavior, criminal behavior, and substance use (Becht et al., 2016; Levey et al., 2019; De Moor et al., 2022). In contrast, individuals who hold a SEM are better able to view their abilities and identity positively and promote self-identity in the face of stressful events (Yeager and Dweck, 2012). Conversely, the avoidant mindset of individuals with a SDM view may lead them to turn away from important opportunities for self-exploration and growth, thereby compromising the development of their self-identity (Kouvelis and Kangas, 2021).

### 1.4 Chain mediation of cognitive reappraisal and self-identity

Furthermore, an individual’s self-identity as a cause of personal well-being is closely related to cognitive reappraisal (Al-Refae et al., 2021). An experiment showed that individuals who are adept at using cognitive reappraisal strategies can alleviate psychological stress, eliminate psychological barriers, and improve their self-identity (Juang et al., 2016). Another study found that cognitive reappraisal has a significant impact on mental health and can reduce self-esteem, depression, and other negative emotions (Zilverstand et al., 2017). Thus, although less direct evidence exists, it can be inferred that cognitive reappraisal and self-identity may be key variables that chain-mediate the relationship between stress mindset and psychological pain.

### 1.5 The present study

The purpose of this study was to investigate the effects of stress mindset, self-identity, and cognitive reappraisal on the symptoms of psychological pain in medical students and to explore the underlying processes contributing to these effects, providing a basis for the effective improvement of their mental health. We hypothesized that (1) stress mindset negatively predicts psychological pain (H1), (2) cognitive reappraisal mediates the relationship between stress mindset and psychological pain (H2), (3) self-identity mediates the relationship between stress mindset and psychological pain (H3), and (4) cognitive reappraisal and self-identity mediate the relationship between stress mindset and psychological pain (H4).

## 2 Methods

### 2.1 Participants

We recruited 2,637 medical students from five medical schools in Shandong Province between March 2024 and June 2024 through convenience sampling. A total of 2,637 questionnaires were returned, and 2,056 were deemed valid, resulting in an effective recovery rate of 77.96%, which included 1,369 female (66.60%) and 687 male students (33.40%).

### 2.2 Procedure

This study used a cross-sectional design with questionnaires distributed online through a WeChat group. The exclusion criteria were (1) major physical illness, (2) recent medication use, (3) psychological counseling or treatment, and (4) incomplete questionnaire responses. Before formally completing the questionnaire, each subject was required to understand and provide informed consent. All participants were anonymized and the data were kept confidential. The researcher responsible for administering the measurements and guiding participants through the questionnaire held a doctoral or master’s degree in psychology. Participants were informed that they could withdraw from the study at any time if they felt uncomfortable while completing the questionnaire. All study procedures were approved by the Ethics Committee of Shandong Second Medical University (IRB number: 2024 YX117).

### 2.3 Instruments

#### 2.3.1 Psychological pain

The Psychache Scale, developed by Holden et al. (2001), was used to measure the degree of psychological pain. The scale was revised by Youfeng (2008) and the revised scale has demonstrated good reliability and validity among college students (Youfeng, 2008). The scale consists of 13 items scored on a 5-point scale (1–5), with a higher total score indicating a greater degree of psychological pain. It includes questions such as “I am suffering because I feel empty.” The internal consistency coefficient of the scale was 0.98 in this study.

#### 2.3.2 Self-identity

Ochse and Plug developed the first self-identity questionnaire based on a Self-Identity Scale derived from Erikson’s theory (Ochse and Plug, 1986). Li and Lou (2009) translated and tested the questionnaire for reliability. The questionnaire included 19 items comprising 12 reverse-scored questions and seven forward-scored questions. A 4-point scale was used (1 = very nonconforming, 4 = very conforming). An example of a question included, “I am proud to be part of the community in which I live.” A higher total score on the questionnaire indicated a higher sense of self-identity. The Cronbach’s alpha coefficient for this questionnaire was 0.87, indicating good reliability.

#### 2.3.3 Emotion regulation strategies

The Emotion Regulation Questionnaire developed by Gross and John (2003) is divided into two dimensions: cognitive reappraisal and expressive inhibition. It consists of 10 items, of which six relate to cognitive reappraisal and four to expressive inhibition. A 7-point scale was used (1 - not at all, 7 - completely), including questions such as, “I often experience more positive emotions (e.g., joy or happiness) by changing the way I think about things.” The scale reflects the individual’s use of cognitive reappraisal and expressive inhibition as strategies for coping with different emotions in daily life. The higher the score, the more frequently the emotion regulation strategies were used. This study used the Cognitive Reappraisal subscale from the scale. This subscale has good reliability and validity, with the internal consistency coefficient of this questionnaire being 0.85 in this study.

#### 2.3.4 Stress mindset

The Stress Mindset Measure (SMM) was developed by Crum et al. to measure people’s perceptions of the effects of stress, such as “Experiencing stress helps me to learn and grow” and “Experiencing stress depletes my health and vigor (Crum et al., 2013).” The scale consists of eight items, and participants are asked to rate each item on a five-point scale (0 = strongly disagree, 4 = strongly agree). The items in the negative affect dimension of stress were reverse scored, and the average score of all items was calculated. A higher score indicated that the individual was more inclined to perceive the effects of stress as positive. The internal consistency coefficient of the questionnaire was 0.76.

### 2.4 Data analysis

**Figure 1:**
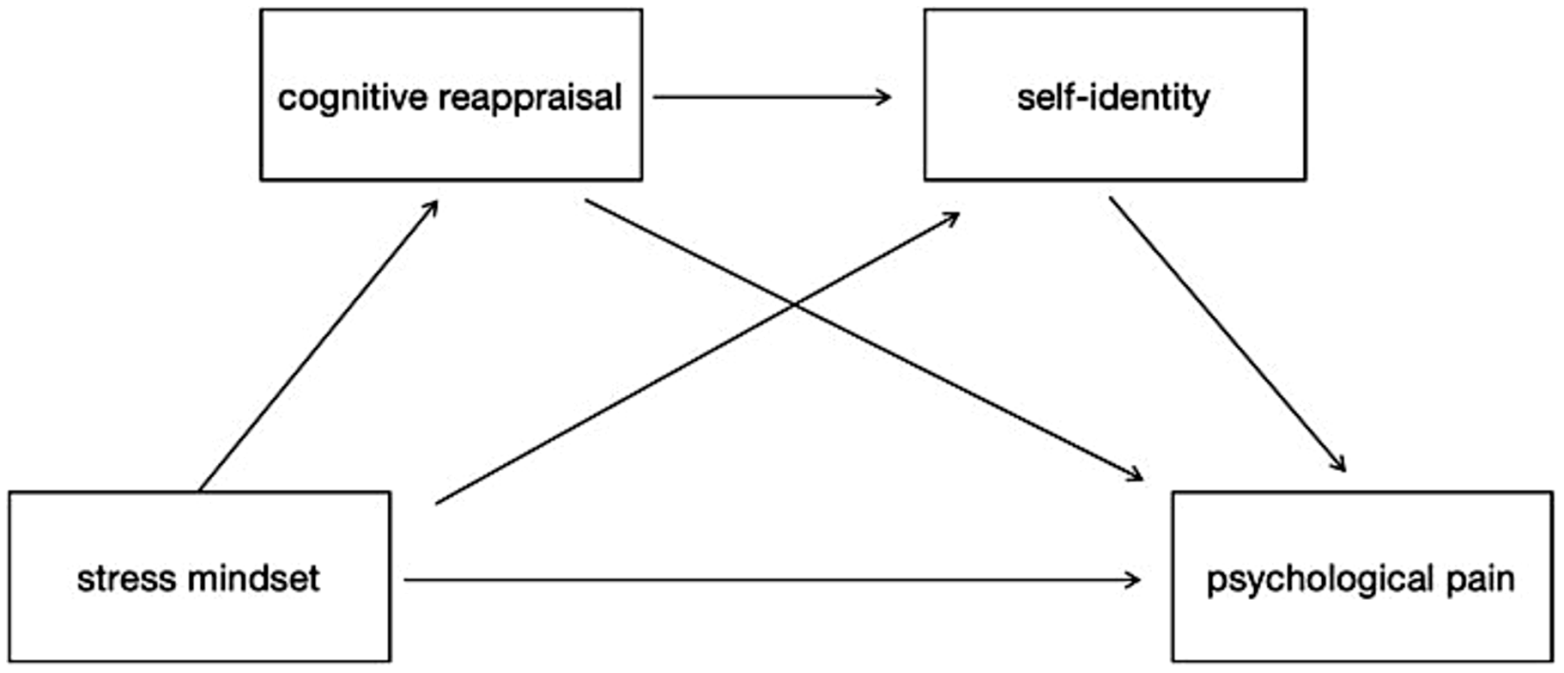
The effect of stress mindset on psychological pain: the chain mediating role of cognitive reappraisal and self-identity.

First, a Harman one-way test was conducted on the sample data using SPSS 29.0 to detect the effect of common method bias. Second, descriptive statistics and correlations were calculated using SPSS 29.0 (Podsakoff et al., 2003). Third, after converting the raw scores of stress mindset, self-identity, cognitive reappraisal, and psychological pain to *Z*-scores, and controlling for gender, the study utilized Mplus 8.3 to test the hypotheses. (Figure 1; Kelloway, 2014). A bootstrap program was employed to assess the size and confidence interval of the indirect effects, generating 10,000 bootstrap samples from the original dataset using random sampling.

## 3 Results

### 3.1 Test of common method bias

The Harman factor test (Westreich, 2012) was used to assess common method bias. Eight common factors were analyzed, with values above 1. The first common factor accounted for only 28.32% of the total variance, which was below the threshold of 40%, indicating that the common method bias was not a significant factor in our study.

### 3.2 Descriptive statistics and correlation analysis

The means, standard deviations, and Pearson’s correlations between the study variables are presented in Table 1. Stress mindset was positively correlated with self-identity (*r* = 0.38, *p* < 0.01) and cognitive reappraisal (*r* = 0.22, *p* < 0.01) but negatively correlated with psychological pain (*r* = −0.30, *p* < 0.01). Self-identity was positively correlated with cognitive reappraisal (*r* = 0.37, *p* < 0.01) and negatively correlated with psychological pain (*r* = −0.58, *p* < 0.01). Cognitive reappraisal was negatively correlated with psychological pain (*r* = −0.28, *p* < 0.01). Thus, Hypothesis 1 was supported.

**Table 1:** Descriptive statistics and correlations among variables (*n* = 2056).

Variables	*M*	SD	1	2	3	4
1 Stress mindset	18.26	4.84	1			
2 Self-identity	51.97	11.59	0.38**	1		
3 Cognitive reappraisal	30.60	6.51	0.22**	0.37**	1	
4 Psychological pain	18.13	8.96	−0.30**	−0.58**	−0.28**	1

*M*, Mean; SD, Standard deviation. ** *p* < 0.01.

### 3.3 The chain mediating roles of cognitive reappraisal and self-identity

**Figure 2:**
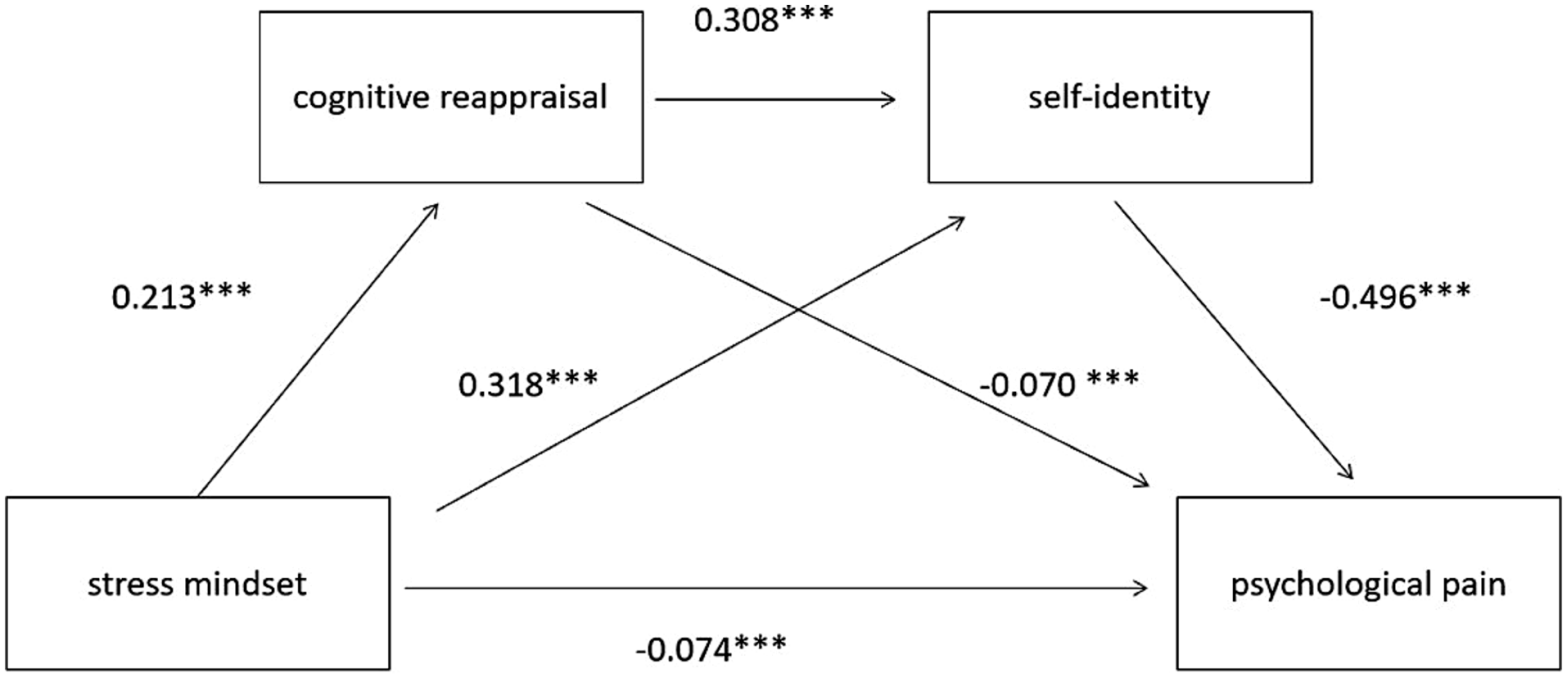
The effect of stress mindset on psychological pain: the chain mediating role of cognitive reappraisal and self-identity.

With stress mindset as the independent variable; psychological pain as the dependent variable; cognitive reappraisal and self-identity as mediating variables; and gender as controlling variables, a chain mediation model was established. The model fitting results confirmed model saturation (c2/df = 0, CFI = 1.00, TLI = 1.00). Specifically, stress mindset was strongly and positively related to cognitive reappraisal (*β* = 0.21, *p* < 0.001), whereas cognitive reappraisal was strongly and negatively related to psychological pain (*β* = −0.07, *p* < 0.001). Further, the 95% confidence interval for the indirect effect value of this mediating path does not include 0, indicating cognitive reappraisal significantly mediated the relationship between stress mindset and psychological pain (indirect effect = −0.02, 95% CI = −0.02–−0.01). As a result, Hypothesis 2 was confirmed. Stress mindset positively predicted self-identity (*β* = 0.32, *p* < 0.001). Self-identity, in turn, negatively predicted psychological pain (*β* = −0.50, *p* < 0.001). The 95% confidence interval for the indirect effect value of this mediating path does not include 0, indicating Self-identity mediated the relationship between stress mindset and psychological pain (indirect effect = −0.16, 95% CI = −0.18–−0.14). As a result, Hypothesis 3 was confirmed. The mediating effects of cognitive reappraisal and self-identity on stress mindset and psychological pain were significant (indirect effect = −0.03, 95% CI = −0.04 to-0.03). Stress mindset had a direct relationship with psychological pain (*β* = −0.07, *p* < 0.001). The cognitive reappraisal and self-identity play an incomplete chain mediating role between stress mindset and psychological pain. The details are shown in Tables 2, 3 and Figure 2. Consequently, Hypothesis 4 was confirmed.

**Table 2:** Path analysis results (*N* = 2,056).

Dependent variable	Independent variable	Estimate	S.E.	95% CI (Bootstrap)		*p*-value
				Lower	Upper	
Psychological pain	ON					
	Cognitive reappraisal	−0.07	0.02	−0.11	−0.03	0.000
	Self-identity	−0.50	0.02	−0.54	−0.45	0.000
	Stress mindset	−0.07	0.02	−0.11	−0.04	0.000
	Gender	−0.08	0.04	−0.16	−0.01	0.026
Cognitive reappraisal	ON					
	Stress mindset	0.21	0.02	0.17	0.26	0.000
	Gender	−0.09	0.05	−0.18	−0.01	0.043
Self-identity	ON					
	Stress mindset	0.32	0.02	0.28	0.36	0.000
	Cognitive reappraisal	0.31	0.02	0.27	0.35	0.000
	Gender	0.05	0.04	−0.04	0.13	0.257

**Table 3:** Direct and indirect effects of the serial mediation model.

Model path	Estimate	S.E.	95% CI (Bootstrap)		*p*-value
			Lower	Upper	
Ind1	−0.02	0.00	−0.02	−0.01	0.001
Ind2	−0.16	0.01	−0.18	−0.14	0.000
Ind3	−0.03	0.00	−0.04	−0.03	0.000
Direct effect	−0.07	0.02	−0.11	−0.04	0.000
Total	−0.28	0.02	−0.32	−0.24	0.000

Ind1, stress mindset → cognitive reappraisal → psychological pain.

Ind2, stress mindset → self-identity → psychological pain.

Ind3, stress mindset → cognitive reappraisal → self-identity → psychological pain.

## 4 Discussion

Psychological pain is a common phenomenon worldwide that has many adverse effects on personal development. This study explored the potential relationships among stress mindset, self-identity, cognitive reappraisal, and psychological pain. Additionally, based on implicit theories and emotion regulation theory, we proposed and examined a chain mediation model. The findings suggest that cognitive reappraisal and self-identity play independent or chain mediating roles between stress mindset and psychological pain. These results contribute to a better understanding of the possible links between stress mindset and psychological pain.

### 4.1 Relationship between stress mindset and psychological pain

Consistent with Hypothesis 1, our findings indicate that a stress mindset may be associated with psychological pain. Specifically, medical students with a SEM reported lower levels of psychological pain, whereas those with a SDM reported higher levels. This result aligns with previous findings suggesting that an individual’s mindset or perception of stress may influence their psychological well-being (Chen and Qu, 2021). One possible explanation is that medical students with a SEM are more likely to perceive stress as a challenge rather than a threat, potentially reducing their fear of stressors and thereby mitigating psychological pain (Horiuchi et al., 2018). These findings contribute to the growing body of literature on the relationship between stress mindset and psychological pain.

### 4.2 Mediating role of cognitive reappraisal

Consistent with Hypothesis 2, the results suggest that cognitive reappraisal may play a mediating role in the relationship between stress mindset and psychological pain. In the first part of the mediation process, stress mindset was associated with cognitive reappraisal, which is in line with previous studies (Crum et al., 2017). One possible explanation is that a positive stress mindset may encourage individuals to adopt more adaptive coping strategies and reduce avoidance of negative emotions, thereby facilitating cognitive reappraisal. Additionally, cognitive reappraisal was negatively associated with psychological pain, a finding that aligns with prior research (Meeus et al., 2010; Hatano et al., 2018). This may suggest that individuals who engage in cognitive reappraisal are better able to manage negative emotions, which could reduce the impact of these emotions on their psychological well-being. Overall, these findings highlight the potential role of cognitive reappraisal in alleviating psychological pain.

### 4.3 Mediating role of self-identity

Consistent with Hypothesis 3, self-identity appeared to serve as an important explanatory pathway linking stress mindset and psychological pain. Specifically, medical students with a SEM were more likely to report a stronger sense of self-identity, which was, in turn, associated with lower levels of psychological pain. This finding aligns with previous research suggesting that self-identity may mediate the relationship between stress and mental health (Thoits, 2013). In the first part of the mediation process, stress mindset was positively related to self-identity, consistent with studies indicating that stress mindset can influence cognition (Crum et al., 2018). One possible interpretation is that individuals with a SEM tend to view stress as a challenge, which may help them build resilience and adaptability when facing difficulties. This adaptive perspective might foster a stronger sense of self-identity (Ewert et al., 2024). In the second part of the mediation process, self-identity was negatively associated with psychological pain, supporting studies indicating that individuals with high self-identity tend to have better psychological well-being (Ewert et al., 2024). This evidence suggests a link between self-identity and psychological pain.

### 4.4 The chain mediating roles of cognitive reappraisal and self-identity

Consistent with Hypothesis 4, the study findings suggest that cognitive reappraisal and self-identity may act as sequential mediators in the relationship between stress mindset and psychological pain. These findings align with prior research suggesting that cognition reappraisal is a positive emotion regulation strategy that involves the reinterpretation of situations (Keech et al., 2021). Through cognitive reappraisal, individuals may be more likely to perceive stress as a challenge rather than a threat, which could, in turn, contribute to a stronger sense of self-identity (Goldin et al., 2021). Moreover, prior studies indicate that individuals who frequently engage in cognitive reappraisal may experience fewer negative emotions, potentially facilitating the maintenance of self-identity (Tugade and Fredrickson, 2004). Based on these findings, it is plausible that stress mindset influences psychological pain through cognitive reappraisal and self-identity.

### 4.5 Limitations

There are several limitations in this study that must be acknowledged. First, the gender imbalance in our sample may limit the generalizability of the findings, as the proportion of female participants (66.60%) was higher than that of male participants (33.40%). While some studies suggest that gender may not have a significant effect on stress mindset and psychological pain, a more balanced sample in future research would help validate and extend our results (Calati et al., 2022; Čopec et al., 2022).

Second, psychological pain is influenced by multiple factors, including social support and resilience, which were not directly examined in this study. Social support, encompassing both perceived and actual instrumental or emotional assistance from social networks, communities, and close relationships that plays a crucial role in buffering stress and enhancing psychological well-being (La Buissonnière-Ariza et al., 2018). Future studies could explore its potential mediating or moderating effects in the relationship between stress mindset and psychological pain. Similarly, resilience, defined as an individual’s ability to adapt and recover in the face of adversity, has been shown to mitigate psychological pain(Özkan and Karakaya, 2025). Incorporating these variables in future research could provide a more comprehensive understanding of the factors contributing to psychological pain.

Third, due to the cross-sectional nature of this study, causal relationships cannot be established. The observed associations should therefore be interpreted as heuristic rather than definitive pathways. To better clarify the causal effects of stress mindset on psychological pain, future research should employ longitudinal designs with systematic data tracking, such as regular long-term follow-ups with participants. This would provide more robust evidence on how stress mindset influences psychological pain over time and offer valuable insights into potential intervention strategies.

### 4.6 Implications

Given the potential role of stress mindset, cognitive reappraisal, and self-identity in alleviating psychological pain among medical students, the findings of this study provide new insights into clinical interventions.

First, Cognitive Behavioral Therapy (CBT) has been widely used to treat anxiety and depression (Cuijpers et al., 2019). Given the critical role of stress mindset in psychological pain, our study suggests that clinical practice could incorporate strategies to modify individuals’ stress mindsets within the CBT framework. This approach may foster adaptive psychological coping mechanisms and ultimately mitigate psychological pain.

Second, cognitive reappraisal plays a crucial role in regulating psychological pain. Integrating cognitive reappraisal techniques into clinical interventions, such as Emotion-Focused Therapy (EFT), could help individuals enhance their stress management skills and reduce the adverse effects of psychological pain.

Furthermore, self-identity is a key factor in psychological well-being. Studies have shown that a lack of self-identity may exacerbate psychological pain. Therefore, interventions focused on fostering self-identity development, such as Humanistic Therapy or Narrative Therapy, may have positive effects on alleviating psychological pain. For instance, clinical interventions could guide individuals in exploring their personal values, constructing meaning, and strengthening their professional identity, thereby enhancing psychological resilience (Mahmoud and Rothenberger, 2019).

Moreover, from an educational and preventive perspective, institutions should recognize the importance of early identification and intervention in psychological pain through mental health education, group counseling, and stress management training. These approaches could be essential in reducing psychological pain among medical students.

In conclusion, effectively addressing psychological pain in medical students requires a comprehensive, multi-level intervention strategy that considers stress mindset, cognitive reappraisal, and self-identity. The findings of this study suggest a novel direction for clinical practice by integrating self-mindset regulation with psychotherapeutic interventions. This approach not only targets the symptoms of psychological pain but also incorporates potential protective factors that may alleviate its impact.

## Data Availability

The raw data supporting the conclusions of this article will be made available by the authors, without undue reservation.
